# Cavernous sinus involvement is not a risk factor for the primary tumor site treatment outcome of Sinonasal adenoid cystic carcinoma

**DOI:** 10.1186/s40463-018-0257-z

**Published:** 2018-02-05

**Authors:** Yi-Chan Lee, Ta-Jen Lee, Ngan-Ming Tsang, Yenlin Huang, Cheng-Lung Hsu, Li-Jen Hsin, Yi-Hsuan Lee, Kai-Ping Chang

**Affiliations:** 1 Department of Otolaryngology - Head and Neck Surgery, Chang Gung Memorial Hospital, Keelung, Taiwan; 2 Department of Otolaryngology - Head and Neck Surgery, Chang Gung Memorial Hospital, Taoyuan, Taiwan; 3 Department of Radiation Oncology, Chang Gung Memorial Hospital, Taoyuan, Taiwan; 4 Department of Hematology-Oncology, Chang Gung Memorial Hospital, Taoyuan, Taiwan; 5 Department of Pathology, Chang Gung Memorial Hospital, Taoyuan, Taiwan; 6 Department of Orthopedic Surgery, Buddhist Tzu-Chi General Hospital, Taipei, Taiwan; 7 College of Medicine, Chang Gung University, Taoyuan, Taiwan

**Keywords:** Sinonasal cancer, Adenoid cystic carcinoma, Cavernous sinus, Head and neck, Surgery, Chemoradiation

## Abstract

**Background:**

Sinonasal adenoid cystic carcinoma is a rare malignancy of the head and neck. Cavernous sinus invasion from sinonasal adenoid cystic carcinoma and its related management have rarely been investigated. This study evaluated the relationship between treatment outcome and cavernous sinus involvement in addition to other parameters.

**Methods:**

A retrospective case series study was conducted at a tertiary referral center. The medical records of 47 patients diagnosed with primary sinonasal adenoid cystic carcinoma between 1984 and 2015 were retrospectively reviewed. The survival impact of the primary treatment modalities and the anatomic sites of tumor involvement were analyzed.

**Results:**

Cavernous sinus invasion was observed in 8 patients (17%), of whom 7 had ACC tumors originating from the maxillary sinus. The results of univariate analysis revealed that tumor stage, primary surgery, and the absence of skull-base and infratemporal fossa invasion were associated with better overall survival (*P* = 0.033, *P* = 0.012, *P* = 0.011, and *P* = 0.040, respectively) and better disease-free survival (*P* = 0.019, *P* = 0.001, *P* = 0.017, and *P* = 0.029, respectively). Multivariate analysis identified primary surgery as the only independent prognostic factor for disease-free survival (*P* = 0.026). Cavernous sinus invasion by sinonasal adenoid cystic carcinoma was not associated with worse overall survival or disease-free survival (*P* = 0.200 and *P* = 0.198, respectively).

**Conclusions:**

Because maxillary adenoid cystic carcinoma is associated with a higher rate of cavernous sinus invasion, such cases warrant caution during preoperative planning. Primary surgery as the initial therapy provides better locoregional control and survival for patients with sinonasal adenoid cystic carcinoma. Cavernous sinus invasion did not significantly impact survival; thus, it should not be regarded as a contraindication for curative treatment.

## Background

Sinonasal malignant tumors are rare, accounting for approximately 3% of upper respiratory tract cancers. Adenoid cystic carcinoma (ACC), arising from the salivary glands, is the fourth most common malignant tumor in the sinonasal tract [1, 2]. Approximately 10–25% of ACC tumors in the head and neck region originate in the sinonasal region [3]. Some symptoms of sinonasal ACCs are similar to chronic sinusitis symptoms; thus, the diagnosis of sinonasal ACC is often delayed after initial presentation [3–5].

Previous studies have demonstrated that ACC is an aggressive tumor that spreads through either direct bone invasion or perineural invasion and frequently involves the skull base or intracranial region [3]. Cavernous sinus involvement of sinonasal ACC often occurs via perineural spread [6–10]; however, the incidence and treatment outcomes of cavernous sinus invasion by these tumors have never been addressed or analyzed. Hence, this study investigates the incidence and management of cavernous sinus invasion by sinonasal ACC in a single tertiary referral hospital. The primary outcome of this study is to clarify the prognosis of cavernous sinus involvement in patients with ACC upon presentation. Prognostic factors, treatment modalities, outcomes, and survival of patients with sinonasal ACC tumors were also analyzed.

## Methods

This study enrolled patients who were diagnosed with sinonasal ACC between January 1984 and August 2015 at Chang Gung Memorial Hospital (Linko Medical Center, Taoyuan, Taiwan). Patients with at least one of the following conditions were excluded: prior history of malignancy, presence of a concomitant primary cancer (synchronous or metachronous), recurrent disease, or treatment by another hospital. Patients enrolled in this study had pathologically confirmed ACC tumors at one of the following sites: maxillary sinus, ethmoid sinus, sphenoid sinus, frontal sinus, or nasal cavity. ACC tumors originating from the nasopharynx were also excluded due to differences in staging and anatomical origin. The retrospective review of medical records for this study was approved by the Institutional Review Board (201600713B0).

Patients in the study received standard preoperative work-ups according to institutional guidelines, including a detailed history, complete physical examination, computed tomography or magnetic resonance imaging, chest radiographs, bone scan, and abdominal ultrasonography. Computed tomography or magnetic resonance imaging was used to determine the epicenter of the neoplasm. If the tumor involved multiple sites of the sinonasal region, the site with the greatest tumor volume was considered the epicenter. Tumor, node, and metastasis (TNM) staging was performed using the criteria for nasal cavity and paranasal sinus malignancy in the 2010 American Joint Committee on Cancer Staging Manual.

Medical records were reviewed for information regarding surgery, adjuvant therapy, radiotherapy, chemotherapy, recurrences, and follow-up period. If the primary treatment modality was surgery, the types of surgical resection included septectomy, maxillectomy, craniofacial resection, or endoscopic resection. Adjuvant therapy after surgery included radiotherapy or chemoradiotherapy. Chemoradiotherapy rather than surgical excision was used to treat cavernous sinus involvement. In patients who refused surgery as their primary treatment, radiotherapy (total target dose, at least 60 Gy delivered in fractions of 1.8–2 Gy per day for 5 days per week) was administered with or without concurrent chemotherapy (cisplatin-based regimens) [11]. All of the patients completed regular follow-up visits every 2 months for the first year after discharge, every 3 months for the second year, and every 6 months thereafter.

The associations between different categorical parameters were analyzed using the chi-square test or Fisher’s exact test. All of the surviving patients received follow-ups until November 2016 or the date of death. In terms of patient survival, the overall survival (OS) and disease-free survival (DFS) rates were estimated using the Kaplan-Meier method and compared with the log-rank test. For OS, the event of interest was death from ACC directly or from an unrelated cause. For DFS, the event was death or any tumor relapse occurring loco-regionally or distantly. Multivariate regression analyses were applied to identify independent risk factors for OS and DFS. Statistical analyses were performed using PASW Statistics 18 (SPSS, Chicago, IL, USA). All *P* values were two-sided with the significance level set at *P* < 0.05.

## Results

### Patient characteristics

During the study period, 47 patients (20 females [42.6%] and 27 males [57.4%]) with sinonasal ACC tumors were enrolled (Table 1). The primary sites of sinonasal ACC were the maxillary sinus (*n* = 30), the ethmoid sinus (*n* = 8), and the nasal cavity (*n* = 9). Anatomic sites of ACC involvement were the cavernous sinus (*n* = 8; 17.0%), the skull base (*n* = 11; 23.4%), infratemporal fossa (*n* = 18; 38.3%), and cheek skin (*n* = 7; 14.9%). Of the 8 patients with cavernous sinus invasion, 7 had maxillary and 1 had ethmoidal ACC. Tumor characteristics are described in Table 1.

**Table 1:** Patient demographics and tumor stage

Parameters	N	Percent
All patients	47	100
Sex		
Male	27	57.4
Female	20	42.6
Age (yr)		
Median	52.4	
Range	16.3–81.2	
Follow-up (mo)		
Median	56.0	
Range	3.7–326.5	
Tobacco use		
Never	33	70.2
Former/current	14	29.8
Alcohol use		
Never	36	76.6
Former/current	11	23.4
T stage		
T1	6	12.8
T2	1	2.1
T3	8	17.0
T4a	19	40.4
T4b	13	27.7
N stage		
N0	46	97.9
N1–3	1	2.1
M stage		
M0	46	97.9
M1	1	2.1
Clinical stage		
I	6	12.8
II	1	2.1
III	8	17.0
IVa	18	38.3
IVb	13	27.7
IVc	1	2.1

### Treatment modality

Surgical resection was the initial primary treatment in 30 patients with T1 (*n* = 6), T2 (*n* = 1), T3 (*n* = 7), T4a (*n* = 11), and T4b (*n* = 5) tumors. Of these 30 patients, 3 were treated with surgery alone, whereas the remaining 12 and 15 received adjuvant radiotherapy and chemoradiation therapy, respectively. Either chemoradiation or radiotherapy was the initial primary treatment for the remaining 17 patients with T3 (*n* = 1), T4a (*n* = 8), and T4b (n = 8) tumors. Of these 17 patients, 5 received radiotherapy alone, 11 received chemoradiotherapy, and 1 refused treatment due to his M1 status.

### Tumor relapse

Fourteen patients developed recurrence (29.8%), and the time from initial treatment to recurrence ranged from 15.7 to 257.7 months (median 53.6 months). The recurrence was local in 12 cases (25.5%) and regional in 3 cases (6.4%). Of the 14 patients with locoregional recurrence, 11 (78.6%) had stage III or IV disease at initial presentation.

### Side effects and complications

No severe complications were noted in the 8 patients with cavernous sinus invasion. Among the 8 patients, seven encountered temporary malaise with headache and were treated with dexamethasone. Many of them recovered, while 2 patients had trigeminal neuropathy (common toxicity criteria, grade 2), also regressing gradually with steroid use.

### Factors influencing survival

**Fig. 1:**
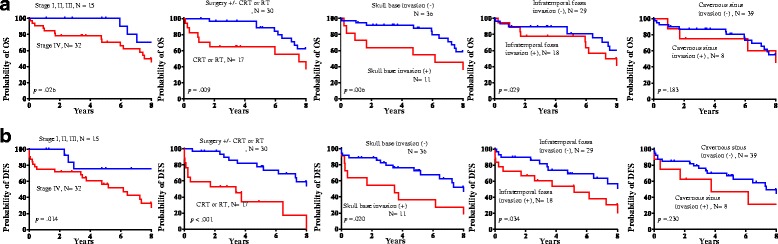
**a** Kaplan-Meier curves of overall survival by tumor stage, primary surgery, skull base invasion, infratemporal fossa invasion and cavernous sinus invasion. **b** Kaplan-Meier curves of disease-free survival by tumor stage, primary surgery, skull base invasion, infratemporal fossa invasion and cavernous sinus invasion. CRT, chemoradiation therapy; RT, radiotherapy

The median time for OS and DFS was 77.1 months and 57.1 months, respectively. At 3, 5, and 10 years, the OS rates were 76.6%, 61.7%, and 23.4%, respectively, and the DFS rates were 66.0%, 44.7%, and 14.9%, respectively. In analyzing factors affecting survival during follow-up, patients with stage IV disease had significantly worse OS and DFS than patients with stage I, II, or III disease (*P* = 0.026 and *P* = 0.014, respectively; Fig. 1a-b). OS and DFS were better in those who received surgery as the initial primary therapy than in those who received radiotherapy or chemoradiotherapy as the initial primary treatment (*P* = 0.009 and *P* < 0.001, respectively; Fig. 1a-b). Skull base invasion and infratemporal fossa invasion were specifically identified as negative factors affecting OS and DFS, whereas cavernous sinus invasion was not (Fig. 1a-b). In Fisher’s exact test, no significant difference was found for either the local recurrence rate or the distant metastasis rate between patients with and without cavernous sinus invasion (*P* = 0.658, *P* = 1.000, respectively).

**Fig. 2:**
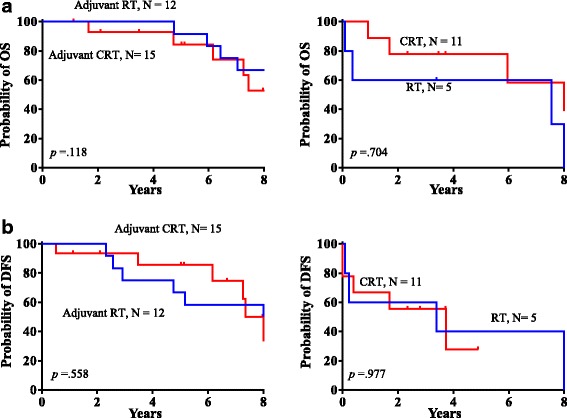
**a** Kaplan-Meier curves of overall survival by postoperative adjuvant therapy and primary chemoradiation therapy compared with primary radiotherapy. **b** Kaplan-Meier curves of disease-free survival by postoperative adjuvant therapy and primary chemoradiation therapy compared with primary radiotherapy. CRT, chemoradiation therapy; RT, radiotherapy

Patients who received surgery as the initial treatment included a group of 12 patients who received postoperative adjuvant radiotherapy and another group of 15 who received postoperative chemoradiation therapy. The differences in OS and DFS between these two groups were not significant (*P* = 0.118 and *P* = 0.558, respectively; Fig. 2a-b). The differences in OS and DFS between patients treated with radiotherapy alone and those treated with chemoradiation therapy alone were also not significant (*P* = 0.704 and *P* = 0.977, respectively; Fig. 2a-b).

To identify independent predictors of survival, multivariate analysis for OS and DFS was performed using age, sex, stage, skull base invasion, infratemporal fossa invasion, and cavernous sinus invasion as parameters in a Cox proportional regression model. Only surgery as the primary treatment was an independent factor influencing DFS (adjusted hazard ratio: 0.280, 95% confidence interval = 0.091–0.857, *P* = 0.026, Tables 2 and 3).

**Table 2:** Multivariate analysis of risk factors in relation to overall survival

	Univariate analysis		Multivariate analysis	
Variables	Hazard ratio (95% CI)	*P*-value	Adjusted hazard ratio (95% CI)	*P*-value
Age (yr)				
≤ 65	1		1	
> 65	1.529 (0.523–4.471)	0.438	2.119 (0.554–8.105)	0.273
Sex				
Female	1		1	
Male	0.863 (0.391–1.905)	0.715	1.284 (0.423–3.899)	0.659
Clinical stage				
I, II, III	1		1	
IV	2.898 (1.088–7.719)	0.033^*^	2.397 (0.612–9.386)	0.209
Surgery				
No	1		1	
Yes	0.358 (0.161–0.794)	0.012^*^	0.690 (0.247–1.927)	0.479
Skull base invasion				
No	1		1	
Yes	3.054 (1.290–7.233)	0.011^*^	2.693 (0.611–11.858)	0.190
Infratemporal fossa invasion				
No	1		1	
Yes	2.414 (1.041–5.600)	0.040^*^	1.500 (0.447–5.027)	0.512
Cavernous sinus invasion				
No	1		1	
Yes	1.874 (0.718–4.893)	0.200	0.541 (0.138–2.128)	0.380

*Abbreviations*: *CI* confidence interval

^*^statistically significant

**Table 3:** Multivariate analysis of risk factors in relation to disease-free survival

	Univariate analysis		Multivariate analysis	
Variables	Hazard ratio (95% CI)	*P*-value	Adjusted hazard ratio (95% CI)	*P*-value
Age (yr)				
≤ 65	1		1	
> 65	1.545 (0.528–4.520)	0.427	1.670 (0.600–4.649)	0.326
Sex				
Female	1		1	
Male	1.036 (0.477–2.250)	0.929	1.841 (0.494–6.857)	0.363
Clinical stage				
I, II, III	1		1	
IV	3.334 (1.217–8.129)	0.019^*^	2.059 (0.527–8.048)	0.299
Surgery				
No	1		1	
Yes	0.231 (0.100–0.536)	0.001^*^	0.280 (0.091–0.857)	0.026^*^
Skull base invasion				
No	1		1	
Yes	2.750 (1.200–6.302)	0.017^*^	1.140 (0.253–5.146)	0.865
Infratemporal fossa invasion				
No	1		1	
Yes	2.251 (1.098–5.789)	0.029^*^	2.259 (0.660–7.734)	0.194
Cavernous sinus invasion				
No	1		1	
Yes	1.845 (0.725–4.693)	0.198	1.083 (0.258–4.539)	0.913

*Abbreviations*: *CI* confidence interval

^*^statistically significant

## Discussion

In previous studies, most cases were predominantly stage IV because of the non-specific symptoms and late presentation of this rare malignancy and were associated with poor survival [3, 12–15]. In this study, stage IV tumors were also diagnosed in most (68.1%) patients, and those with stage IV disease exhibited a significantly worse OS. The primary treatment modalities for the original disease also affected survival outcome. Kaplan-Meier survival analysis showed that primary surgery as the initial treatment provided better OS and DFS, and the results of multivariate analysis indicated an association with a better DFS. A previous study demonstrated that the surgery type (endoscopic or external) was irrelevant if the tumor was completely resected [14]. Kaplan-Meyer analyses assessing the anatomic sites of tumor invasion both in this study and in a previous report found that skull base invasion and infratemporal fossa invasion negatively impacted survival [3, 16]. Overall, for stage IV disease, the primary treatment modality (oncologically sound resection) and the anatomical location of tumor involvement were the major prognostic factors for this disease.

Winslow first referred to the small lateral sellar compartment as the “cavernous sinus.” This pair of dural venous sinuses is a well-vascularized structure in the middle cranial fossa surrounded by the brain parenchyma [17]. The presence of cranial nerves III, IV, and V at the sinus capsule, in addition to cranial nerve VI, the internal carotid artery, and surrounding carotid sympathetic nerves, makes surgery at this location challenging. For malignant tumors involving the cavernous sinus, surgical removal of the cavernous sinus lesion is usually destructive and fraught with complications [18, 19]. Some authors have suggested limited excision of cavernous sinus tumors followed by adjuvant radiotherapy to treat the residual tumor; however, assessment of the oncological results for these treatments is not always feasible, and thus, the functional and oncological assessment of these approaches remains unclear [20, 21]. To avoid postoperative disability, we performed radical resection for sinonasal ACC, except when the tumor was involved in the cavernous sinus, and administered adjuvant chemoradiation therapy to the involved lesion. In addition, in seven of the 8 patients with cavernous sinus invasion, the primary site was the maxillary sinus. This may be due to a tendency for ACC tumors to invade perineurally; thus, the ACC tumors in the maxillary sinus would spread frequently through the maxillary branch of the trigeminal nerve. Consequently, in the pretreatment radiological work-ups for patients with maxillary ACC, extreme caution was advocated when examining the cavernous sinus. Among the 8 patients with cavernous sinus involvement, no recurrence was found. Furthermore, cavernous sinus invasion did not appear to cause tumor relapse or to be a negative prognostic factor in our univariate and multivariate analyses. Based on these findings, we suggest that the primary treatment modality for sinonasal ACC with cavernous sinus invasion, similar to other sinonasal ACC tumors, should be surgery. Cavernous sinus invasion should not be considered a contraindication for surgery even if its involvement is not within the surgical field.

According to the literature reviewed, sinonasal ACC with skull base invasion has been mentioned to have a negative influence on survival [3]. In addition, infratemporal fossa invasion has also been associated with worse survival in the treatment of sinonasal malignancy [16]. Involvement of the cavernous sinus, on the other hand, has never been addressed before. The cavernous sinus is a small and complex anatomic structure with many cranial nerves passing through it, so invasion of the cavernous sinus often occurs due to the perineural spread and subsequently results in the apparent symptoms of cranial nerve palsy. The relatively early presentation of related symptoms may contribute to earlier intervention and management. Therefore, the authors assumed that the tumor burden of cavernous sinus invasion might be not usually too high to have a negative impact on survival.

Many studies have demonstrated that postoperative adjuvant radiotherapy can increase survival [3, 5, 15, 22]; however, chemotherapy has not been effective for treating sinonasal ACC [3, 23, 24]. In this study, we divided the 27 patients who received primary surgery into two groups, a postoperative adjuvant radiotherapy group (*n* = 12) and a postoperative adjuvant chemoradiotherapy group (*n* = 15), and observed no significant differences in DFS and OS between them. We also compared patients who received primary radiotherapy alone (*n* = 5) and patients who received primary chemoradiation therapy (*n* = 11) and found no significant difference in treatment outcomes. Based on these findings, the role of cisplatin-based chemotherapy in managing sinonasal ACC remains unclear and requires further study.

There are limitations in the present study. First, this is a retrospective study of a single tertiary referral center lending to potential bias. The relatively small sample size also renders these results somewhat arbitrary. However, sinonasal ACC is a rare disease entity, and cavernous sinus invasion caused by sinonasal ACC is even rarer. The present study attempted to add information to the current treatment of sinonasal ACC. A much larger study may be required to investigate the role of cavernous sinus invasion and different treatment modalities.

## Conclusions

Cavernous sinus invasion was observed in approximately 17% of our patients with sinonasal ACC, and patients with maxillary ACC had a higher rate of cavernous sinus invasion. We found that survival was significantly improved among patients with sinonasal ACC tumors whose primary treatment was surgery. Although the cavernous sinus lesions in this series were treated solely by chemoradiotherapy, cavernous sinus invasion did not negatively impact survival outcomes in terms of OS and DFS. The role of cisplatin-based chemotherapy remains unclear after this retrospective investigation. The data suggest that primary surgery with adjuvant radiotherapy may provide a better treatment outcome for patients with sinonasal ACC, and cavernous sinus invasion is not a contraindication for curative treatment. Clinicians should remain alert to the possibility of cavernous sinus involvement in the pretreatment work-up and use caution when managing maxillary ACC patients.

## Abbreviations

ACC: Adenoid cystic carcinoma
DFS: Disease-free survival
OS: Overall Survival
TNM: Tumor, nodes, metastasis staging system
